# Blocking of inflammatory heparan sulfate domains by specific antibodies is not protective in experimental glomerulonephritis

**DOI:** 10.1371/journal.pone.0261722

**Published:** 2021-12-23

**Authors:** Jasper J. van Gemst, Nathalie J. H. G. Passmann, Angelique L. W. M. M. Rops, Toin H. van Kuppevelt, Jo H. Berden, Markus A. Loeven, Ton J. Rabelink, Bart Smeets, Johan van der Vlag

**Affiliations:** 1 Department of Nephrology, Radboud Institute for Molecular Life Sciences, Radboud University Medical Center, Nijmegen, The Netherlands; 2 Department of Biochemistry, Radboud Institute for Molecular Life Sciences, Radboud University Medical Center, Nijmegen, The Netherlands; 3 Department of Nephrology and Einthoven Laboratory for Vascular Medicine, Leiden University Medical Center, Leiden, The Netherlands; 4 Department of Pathology, Radboud Institute for Molecular Life Sciences, Radboud University Medical Center, Nijmegen, The Netherlands; University of Patras, GREECE

## Abstract

Glomerulonephritis is an acquired serious glomerular disease, which involves the interplay of many factors such as cytokines, chemokines, inflammatory cells, and heparan sulfate (HS). We previously showed that blocking of inflammatory heparan sulfate domains on cultured glomerular endothelium by specific anti-HS single chain antibodies reduced polymorphonuclear cell (PMN) adhesion and chemokine binding. We hypothesized that injection of anti-HS antibodies in PMN-driven experimental glomerulonephritis should reduce glomerular influx of PMNs and thereby lead to a better renal outcome. In contrast to our hypothesis, co-injection of anti-HS antibodies did not alter the final outcome of anti-glomerular basement membrane (anti-GBM)-induced glomerulonephritis. Glomerular PMN influx, normally peaking 2 hours after induction of glomerulonephritis with anti-GBM IgG was not reduced by co-injection of anti-HS antibodies. Four days after induction of glomerulonephritis, albuminuria, renal function, glomerular hyalinosis and fibrin deposition were similar in mice treated and not treated with anti-HS antibodies. Interestingly, we observed transient effects in mice co-injected with anti-HS antibodies compared to mice that did not receive anti-HS antibodies: (i) a decreased renal function 2 hours and 1 day after induction of glomerulonephritis; (ii) an increased albuminuria after 2 hours and 1 day; (iii) an increased glomerular fibrin deposition after 1 day; (iv) a reduced glomerular macrophage influx after 1 day; (v) a sustained glomerular presence of PMNs at day 1 and 4, accompanied by an increased renal expression of IL-6, CXCL1, ICAM-1, L-selectin, CD11b and NF-κB. The mechanism underlying these observations induced by anti-HS antibodies remains unclear, but may be explained by a temporarily altered glycocalyx and/or altered function of PMNs due to the binding of anti-HS antibodies. Nevertheless, the evaluated anti-HS antibodies do not show therapeutic potential in anti-GBM-induced glomerulonephritis. Future research should evaluate other strategies to target HS domains involved in inflammatory processes during glomerulonephritis.

## Introduction

Glomerular diseases can lead to end stage renal disease and thereby require renal replacement therapy such as dialysis or transplantation [1]. Acute glomerulonephritis is characterized by a rapid glomerular influx of leukocytes that instantly damage the glomerular filtration barrier, which may lead to end stage renal disease [2–5]. Leukocyte migration towards the site of inflammation involves the concerted action of cytokines, chemokines, adhesion molecules and glycosaminoglycans (GAGs) [6–9].

The glomerular endothelial glycocalyx is a thick carbohydrate-rich layer covering the endothelium [1]. The healthy glycocalyx contributes to the filtration barrier and prevents the binding of leukocytes. However, a disturbed/diseased glycocalyx contributes to proteinuria, chemokine binding and leukocyte trafficking since the sequential steps of leucocyte migration and adhesion to sites of inflammation are mediated by the glomerular endothelial glycocalyx [1, 7–12]. The glomerular endothelial glycocalyx contains several glycosaminoglycans (GAGs), such as heparan sulfate (HS), chondroitin sulfate, and hyaluronic acid (or hyaluronan) [1, 8, 13]. HS consists of repeating β(1–4) and α(1–4) linked N-acetylglucosamine and glucuronic acid or iduronic acid disaccharide units that can be sulfated at various positions, and thereby HS is structurally the most heterogeneous member of the GAG family [2, 11, 14, 15]. The heterogeneous nature of HS allows the formation of specific binding sites for various ligands, including chemokines and cell adhesion molecules such as integrins and selectins [6, 8–10, 14]. Consequently, HS seems to be the main GAG mediating endothelial cell-chemokine and leukocyte interactions.

The glycocalyx is postulated to be involved in the development and progression of kidney disease, for which interference with the binding of inflammatory mediators to HS can be a promising therapeutic strategy [1, 2, 10, 16–18]. Since there is no available method yet to sequence full-length HS chains, structure-function studies regarding HS rely on specific anti-HS antibodies and animal models deficient in HS modifying enzymes [16, 19–25]. Previously, we used anti-HS antibodies to demonstrate the increased expression of specific HS domains on glomerular endothelium under inflammatory conditions [9, 16, 18–20, 22]. We also showed that the anti-HS antibodies, specific for these inflammatory HS domains, could inhibit the rolling and firm adhesion of leukocytes to activated glomerular endothelial cells [16]. Subsequently, we showed that endothelial-specific disruption of HS modifying enzyme N-deacetylase/sulfotransferase (NDST-1) reduced PMN influx during anti-GBM-induced glomerulonephritis, whereas silencing of NDST-1 in cultured glomerular endothelial cells reduced L-selectin, CXCL1, CXCL2 and CCL2 binding *in vitro* as well [20]. Taken together, we hypothesized that anti-HS antibodies could reduce glomerular PMN influx in experimental glomerulonephritis, thereby leading to a better renal outcome. In the present study we used a previously established mouse model of anti-GBM-induced glomerulonephritis, which is driven by a peaking glomerular PMN influx 2 hours after injection of rabbit anti-mouse GBM IgG, whereas proteinuria and a decline in renal function manifest during subsequent days [3, 20, 21, 26, 27].

In contrast to our hypothesis, co-injection of anti-HS antibodies specific for inflammatory HS domains did not reduce glomerular PMN influx and had no beneficial effect on the renal outcome of anti-GBM-induced glomerulonephritis.

## Methods

### Isolation and purification of anti-HS scFv antibodies

VSV-tagged anti-HS single chain (scFv) antibodies (Table 1) AO4B08, EW3D10, EW4G2, defining inflammatory HS domains [16, 24, 25] and a control anti-HS scFv antibody HS4C3, which detects a non-inflammatory HS domain [23], were produced in *E*.*coli*. Antibodies were purified by cobalt resin affinity purification (Life Technologies, Breda, The Netherlands), followed by characterization of HS binding affinity in ELISA with coated heparan sulfate from bovine kidney (HSBK, Sigma-Aldrich, Zwijndrecht, The Netherlands) and analysis of the purity via SDS-PAGE (Bio-Rad, Veenendaal, The Netherlands) and by Western blotting (Bio-Rad) or coomassie brilliant blue staining (Sigma-Aldrich, Zwijndrecht, The Netherlands) as described previously [28]. Antibodies were concentrated in 10 kDa Amicon centrifuge concentration tubes (Merck chemicals B.V., Amsterdam, The Netherlands).

**Table 1:** Characteristics of HS domains recognized by anti-heparan sulfate (HS) scFv antibodies.

Antibody	V_H_—CDR3 region	HS modifications required for antibody binding:	References
**AO4B08**	SLRMNGWRAHQ	N-sulfation, 6-O sulfation, 2-O sulfation, C5-epimerization	[24]
**EW3D10**	GRTVGRN	N-sulfation, 6-O sulfation	[25]
**EW4G2**	GKVKLPN	N-sulfation, 6-O sulfation, glucuronic acid	[25]
**HS4C3**	GRRLKD	N-sulfation, 6-O sulfation, 3-O sulfation, 2-O sulfation,	[23]

*Given are the amino acid sequence of the VH complementary determining region 3, HS modifications determined to be required for antibody binding, and references.

### Induction of anti-GBM glomerulonephritis in mice with or without co-injection of anti-HS scFv antibodies and determination of albuminuria and blood urea nitrogen concentration

C57bl/6-J Jax mice (Charles River (Leiden, The Netherlands) were housed under pathogen-free conditions and in a temperature-controlled room with a 12-hour light/dark cycle with *ad libitum* access to food and water. Mice were age and gender matched. Group size was determined by a power calculation; assuming a power of 0.9 and alpha of 0.05 and an expected effect of 25–30% based on our previous *in vitro* studies [16], 4 mice were required per group. Mice were anaesthetized by isoflurane inhalation followed by euthanasia via cervical dislocation. All experiments were approved by the Animal Ethical Committee of the Radboud University Nijmegen (RU-DEC 2013–005). Rabbit anti-mouse GBM antibodies have been raised, purified and characterized, as previously described by Rops et al. [20, 21]. Eight week old mice weeks were injected in the tail vein with 8 mg rabbit anti-mouse GBM IgG alone or in combination with 200 μg of anti-HS scFv antibodies (Table 1) to induce anti-GBM glomerulonephritis as previously described [20, 21]. Control mice were injected with sterile PBS. Mice were sacrificed 2 hours, 1 or 4 days after injection of anti-GBM IgG. Urine was collected directly through bladder puncture (2 hours group), or during the last 18 hours (1 and 4 days group) in metabolic cages. Kidneys were fixed in 10% buffered formalin or snap frozen in liquid nitrogen. Urinary albumin concentration was measured by radial immunodiffusion (Mancini). Urinary creatinine and blood urea nitrogen (BUN) concentrations were determined in our diagnostics facility.

### Immunofluorescence staining

Frozen renal cortex sections (2 μm) were fixed in ice-cold acetone for 10 minutes and incubated with primary antibodies diluted in PBS containing 1% bovine serum albumin and 0.05% sodium azide (IF-buffer) for 60 minutes. Directly labeled antibodies included goat anti-mouse C3c- and fibrinogen-fluorescein isothiocyanate (FITC) (Nordic, Tilburg, The Netherlands), goat anti-rabbit IgG Alexa-488, donkey anti-goat IgG Alexa-594 (Life technologies, Breda, The Netherlands), goat anti-Armenian hamster IgG Cy3 (Jackson ImmunoResearch Laboratories, West Grove, PA) and rat anti-mouse GR-1 (RB6.8C5)-FITC (BD Biosciences, Alphen aan de Rijn, The Netherlands). Unlabeled primary antibodies included CD68 (MCA1957; Serotec, Oxford, UK), goat anti-VSV-G protein (Novus Biologicals, Cambridge, UK) and hamster anti-mouse Agrin (MI91) (Dept. of Nephrology, Nijmegen, NL). Sections were postfixed with 1% paraformaldehyde in PBS and embedded in VectaShield mounting medium H-1000 (Brunschwig Chemie, Amsterdam, The Netherlands). Goat anti-rabbit IgG, goat anti-mouse C3c, and fibrinogen staining intensities were scored semi-quantitatively on blinded sections from 0 (no staining) to 10 (100% staining intensity inside the glomeruli) independently by two researchers and averaged over 50 glomeruli. Glomerular influx of PMNs was quantified by counting the number of cells per 50 glomeruli.

### Renal histology

Histological assessment of the kidneys was performed on 4μm thick paraffin sections that were stained using periodic acid-Schiff (PAS) reagent. Slide digitization was performed using a PANNORAMIC 1000 digital slide scanner (3DHistech, Budapest, Hungary) with a 20x objective. The whole slide images (WSI) were analyzed using the Caseviewer 2.4 software (3DHistech, Budapest, Hungary). The histology of all glomeruli in a single kidney cross-section (minimal 63 glomeruli) was evaluated in a blinded manner. The percentage of affected glomeruli, showing thrombosis and/or hyalinosis within the glomerular capillaries, was scored. In addition, it was assessed if the affected glomeruli were only partly affected (≤50% of the glomerular tuft area) or more globally (>50% the glomerular tuft area).

### RNA isolation, reverse transcription and quantitative real-time PCR

RNA was isolated from renal cortex using the RNeasy mini kit (Qiagen, Venlo, The Netherlands) according to manufacturer’s procedures with addition of a DNase-I digestion step. Reverse transcription of 1 μg RNA was performed with RevertAID First Strand cDNA Synthesis kit (Thermo Fisher Scientific, Rockford, USA) according to manufacturer’s procedures. One tenth of cDNA was used as template in quantitative real-time PCR using FastStart SYBR Green Master mix (Roche Diagnostics, Almere, the Netherlands) with gene-specific primers (10 μM; Biolegio, Nijmegen, The Netherlands; Table 2) on the CFX96 PCR system (Bio-Rad). Gene expression levels were quantified by the delta-delta C_T_ method using glyceraldehyde-3-phosphate dehydrogenase (GAPDH) as the housekeeping gene.

**Table 2:** Primer sequences used in quantitative real-time PCR.

Gene	Primer sequence (5’-3’)
**GAPDH**	(F) GTGTTCCTACCCCCAATGTGT C
	(R) GGTCCTCAGTGTAGCCGAAGAT
**IL-6**	(F) TTCCTCTCTGCAAGAGACT
	(R) TGTATCTCTCTGAAGGACT
**CXCL1**	(F) ATAATGCCCTTTTACATTCTTTAAC
	(R) AGTCCTTTGAACGTCTCTGTCC
**ICAM**	(F) GTCGAAGGTGGTTCTTCTGAG
	(R) TCCGTCTGCAGGTCATCTTAGG
**L-selectin**	(F) AATAACGTCAAGTCCTCCCG
	(R) TTAATGGGATGAATGAGCGA
**NF-κB**	(F) CTATGGCTCAGGTGCAGTGT
	(R) TTAATGACAGCAGGAACCCA
**CD11b**	(F) GAACCAGCTTCAGGAAAAG
	(R) GCAAGGGACCAT TAGAGG

### Statistical analysis

Values are expressed as means ± S.E.M. and significance between anti-GBM or anti-GBM + scfV treated mouse groups versus the control group or anti-GBM + scfV groups versus anti-GBM alone was evaluated by ANOVA in combination with Dunnett’s test to correct for multiple comparisons. Significance between two groups was evaluated by student-T-test using GraphPad Prism, version 8.4 software (GraphPad Software, Inc., San Diego, CA).

## Results

### Anti-HS antibodies are detectable in mice glomeruli 2 hours after co-injection with anti-GBM IgG and do not interfere with induction of anti-GBM-induced glomerulonephritis

**Fig 1:**
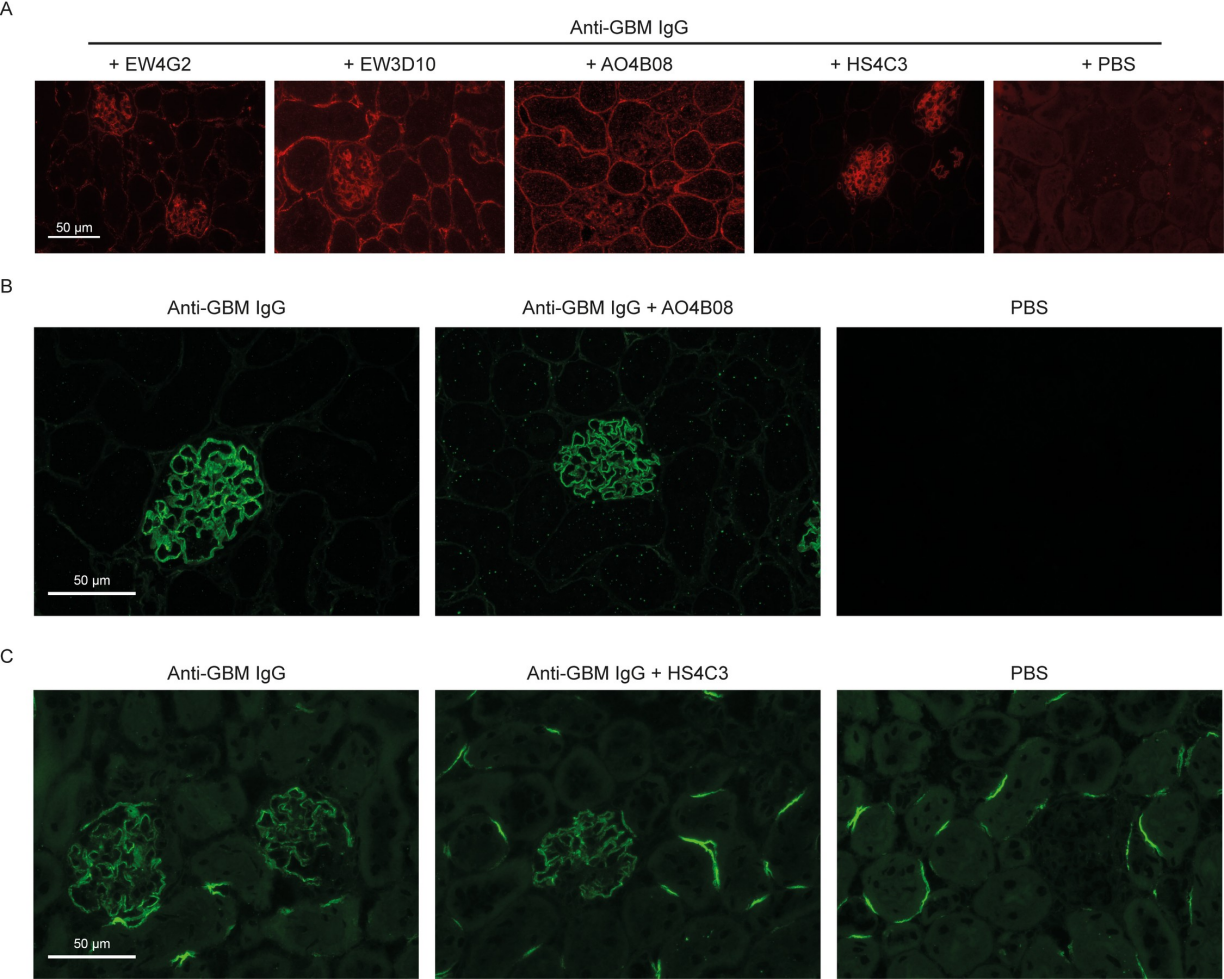
Detection of injected anti-HS antibodies, rabbit IgG and complement C3c in mouse glomeruli after anti-GBM-induced glomerulonephritis. Representative immunofluorescence stainings for (A) anti-HS antibodies with anti-VSV in glomeruli of mice co-injected with anti-GBM IgG serum and anti-HS antibodies, 2 hours after injection (40x magnification), (B) Staining for rabbit IgG in glomeruli of mice co-injected with anti-GBM IgG and anti-HS antibody AO4B08, 2 hours after injection (40x magnification), and (C) Staining for complement C3c in glomeruli of mice co-injected with anti-GBM IgG and anti-HS antibody HS4C3, 2 hours after injection (40x magnification).

To evaluate the effect of anti-HS antibodies on *in vivo* PMN trafficking we employed the well-established anti-GBM glomerulonephritis model, as previously detailed by Rops et al. [20, 21]. This model is PMN-driven and characterized by a heterologous phase, in which rabbit anti-mouse GBM IgG induces a rapid glomerular influx of PMNs that peaks at 2 hours after injection. The autologous phase is starting around day 4, in which newly developed mouse anti-rabbit IgG contribute to the disease as well, however the severity of disease is driven by the initial glomerular influx of PMNs. We first addressed whether the anti-HS antibodies specific for inflammatory HS domains reached the glomerular compartment after injection. Analysis revealed that the administered VSV-tagged anti-HS antibodies indeed were present in the glomeruli of injected mice 2 hours after injection (Fig 1A). Notably, we could not detect anti-HS antibodies in urine using a specific ELISA.

The anti-GBM model is characterized by a rapid linear deposition of injected anti-GBM IgG to the glomerular capillary wall, accompanied by immediate binding of C3c. Injection of rabbit anti-GBM IgG antibodies resulted in a linear binding along the GBM for all time points evaluated, i.e. 2 hours, 1 day and 4 days after induction of glomerulonephritis and no differences were observed when co-injected with anti-HS antibodies (Fig 1B). Furthermore, complement C3c deposition along the GBM was also not affected by co-injection of anti-HS antibodies (Fig 1C). Glomeruli from PBS-injected mice were negative for all stainings (Fig 1A–1C).

### Glomerular PMN and macrophage influx are differentially affected by co-injection of anti-HS antibodies during anti-GBM-induced glomerulonephritis

**Fig 2:**
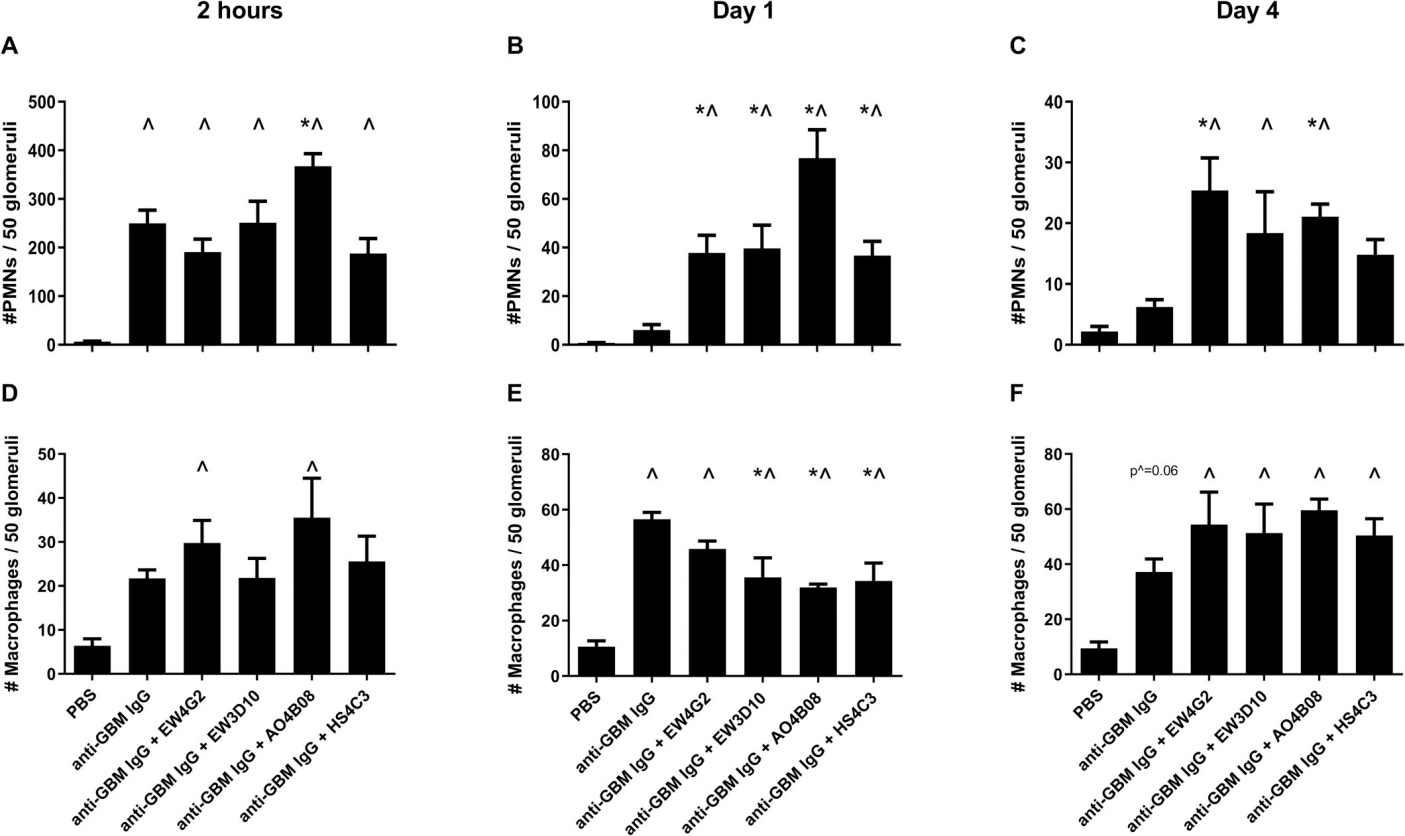
Co-injection of anti-HS antibodies in anti-GBM-induced glomerulonephritis differentially affects glomerular PMN and macrophage influx. Glomerular PMN influx after (A) 2 hours, (B) 1 day and (C) 4 days, and glomerular macrophage influx after (D) 2 hours, (E) 1 day and (F) 4 days analyzed by immunofluorescence staining. Results are expressed as means ± s.e.m. from four mice in each group. ^*P*<0.05 vs. PBS-injected mice. *P<0.05 vs. anti-GBM IgG-injected mice.

PMNs are considered key determinants of glomerular damage and albuminuria during the heterologous phase of anti-GBM-induced glomerulonephritis, and appear to rely on the cytokines of macrophages [3, 29, 30]. Previously, using anti-HS antibodies blocking inflammatory HS domains, we described that these specific HS domains are functionally involved in leukocyte rolling and firm adhesion to the glomerular endothelium *in vitro* [16]. Unexpectedly, our present study shows that co-injection of anti-HS antibodies specific for inflammatory HS domains did not reduce glomerular PMN influx 2 hours after injection of anti-GBM IgG. In contrast, anti-HS antibody AO4B08 co-injection resulted in a significantly increased glomerular PMN influx 2 hours after induction of glomerulonephritis (Figs 2A and S1). Also, 1 day after induction of glomerulonephritis, the number of glomerular PMNs was significantly increased in mice co-injected with all anti-HS antibodies compared to mice that only received anti-GBM IgG (Figs 2B and S1), which persisted to be higher after 4 daysfor mice co-injected with EW4G2 and AO4B08 (Figs 2C and S1). Next we evaluated glomerular macrophage (Mɸ) influx, normally peaking 1 day after anti-GBM-induced glomerulonephritis [3, 31]. Two hours after induction of anti-GBM glomerulonephritis, we observed no significant differences in glomerular macrophage influx (Figs 2D and S2) due to co-injection of anti-HS antibodies, whereas 1 day after induction of anti-GBM glomerulonephritis, co-injection with anti-HS antibodies EW3D10, AO4B08 and HS4C3 significantly reduced glomerular macrophage influx compared to mice that only received anti-GBM IgG (Figs 2E and S2). In contrast, 4 days after induction of anti-GBM glomerulonephritis, co-injections with anti-HS antibodies tended to increase glomerular macrophage influx, compared to anti-GBM IgG injections alone (Figs 2F and S2).

In summary, co-injection of anti-HS antibodies specific for inflammatory HS domains did not reduce glomerular PMN influx in anti-GBM-induced glomerulonephritis, although the kinetics of glomerular PMN and macrophage influx was altered.

### Co-injection of anti-HS antibodies is not protective during anti-GBM-induced glomerulonephritis, but rather transiently increases albuminuria and reduces renal function

**Fig 3:**
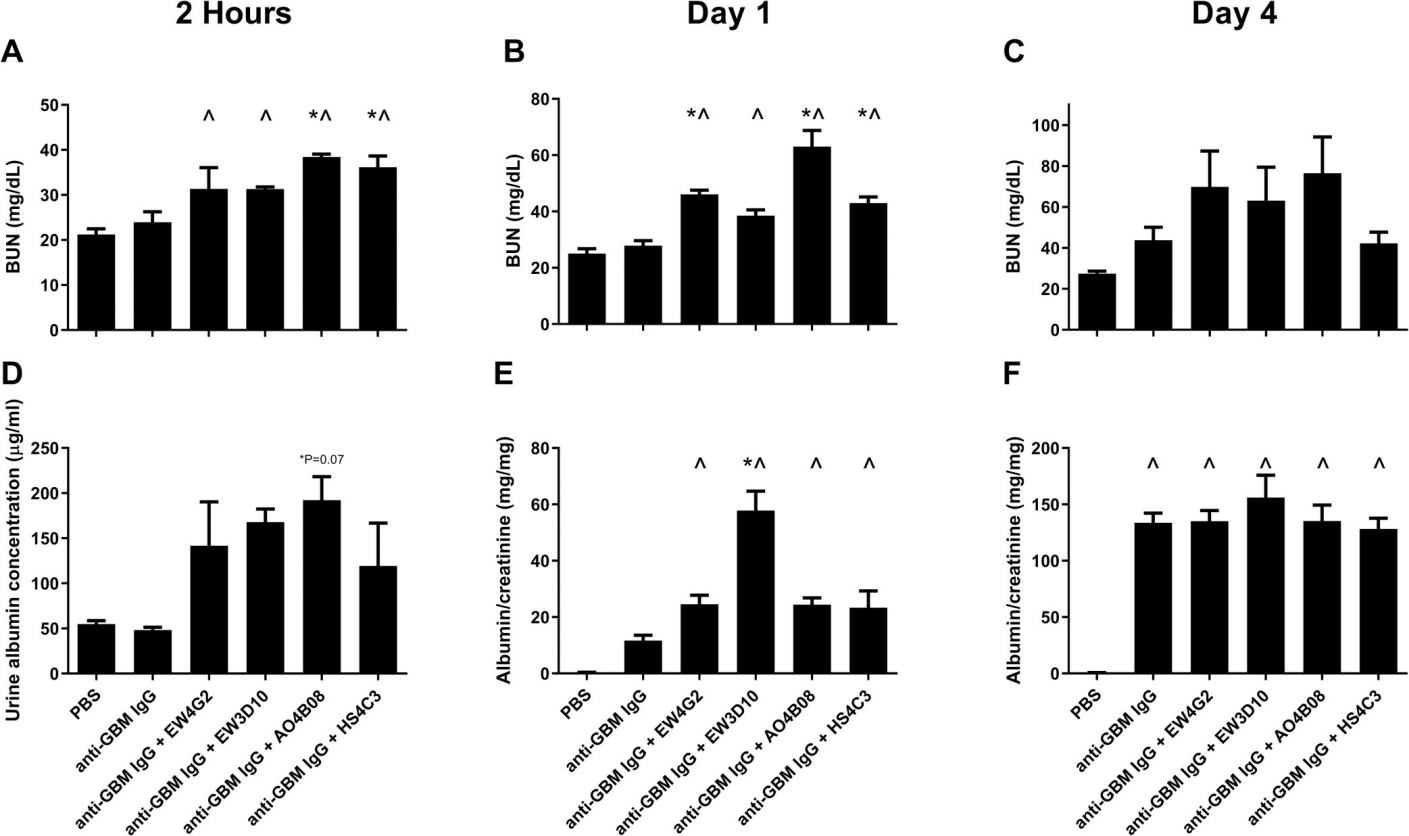
Co-injection of anti-HS antibodies during anti-GBM-induced glomerulonephritis induces a transient increase in albuminuria and decline in renal function. Blood urea nitrogen (BUN) levels after (A) 2 hours, (B) 1 day and (C) 4 days. Urinary albumin concentration after 2 hours (D) and urinary albumin:creatinine ratios after (E) 1 day and (F) 4 days anti-GBM-induced glomerulonephritis. Results are expressed as means ± s.e.m. from four mice in each group. ^*P*<0.05 vs. PBS-injected mice. *P<0.05 vs. anti-GBM IgG-injected mice.

In accordance with our previous findings, two hours after induction of anti-GBM glomerulonephritis we did not observe albuminuria nor increased BUN concentrations (a measure for renal function) in mice that only received anti-GBM IgG, whereas 1 and 4 days after induction of anti-GBM glomerulonephritis, respectively, albuminuria increased and renal function started to decline (Fig 3) [20, 21]. Interestingly, already 2 hours and 1 day after induction of anti-GBM glomerulonephritis, mice co-injected with anti-HS antibodies specific for inflammatory HS domains as well as the anti-HS antibody HS4C3 specific for a non-inflammatory HS domain, displayed an increased albuminuria and/or reduced renal function, compared to mice injected only with anti-GBM IgG (Fig 3A, 3B, 3D and 3E). However, 4 days after induction of anti-GBM glomerulonephritis there was no significant difference in albuminuria and renal function for mice co-injected with anti-HS antibodies or anti-GBM IgG alone (Fig 3C and 3F). In summary, co-injection of anti-HS antibodies induced a transient increase in albuminuria and decrease in renal function, whereas 4 days after induction of anti-GBM glomerulonephritis, albuminuria and renal function were similar to mice that only received anti-GBM IgG.

### Anti-HS antibody co-injections result in an accelerated glomerular fibrin deposition during anti-GBM-induced glomerulonephritis, but a similar fibrin deposition and hyalinosis after 4 days

**Fig 4:**
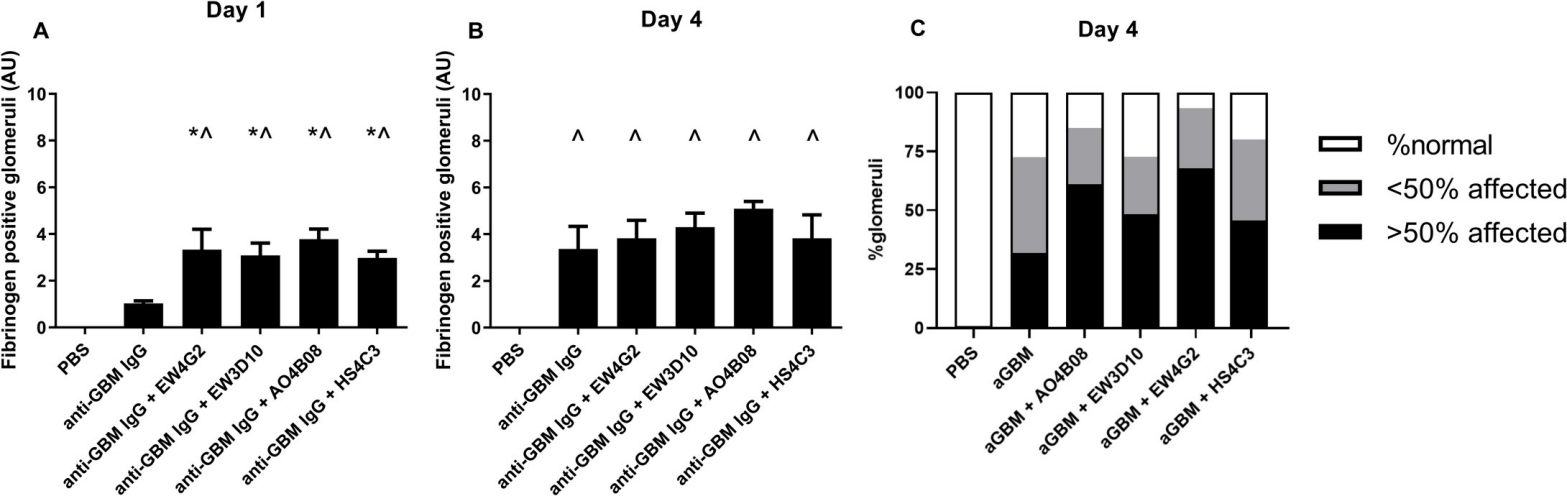
Co-injection of anti-HS antibodies increases fibrin deposition 1 day after induction of anti-GBM glomerulonephritis. Glomerular fibrin deposition after (A) 1 day and (B) 4 days of anti-GBM IgG-induced glomerulonephritis, analyzed by immunofluorescence staining and scored semi-quantitatively from 0–10 based on the percentage of glomeruli with positive staining. Glomerular lesions, identified as thrombosis and hyalinosis was scored on PAS stained renal sections for at least 63 glomeruli per mouse 4 days after injection with PBS, anti-GBM IgG alone or in combination with anti-HS antibodies as normal, containing <50% affected or >50% affected (C). Results are expressed as means ± s.e.m. from four mice in each group. ^*P*<0.05 vs. PBS-injected mice. *P<0.05 vs. anti-GBM IgG-injected mice.

Substantial fibrin deposition and hyalinosis is normally observed 4 days after anti-GBM-induced glomerulonephritis and correlates with, and might thus be an indication for, the degree of glomerular damage. Previous research has shown a direct correlation between glomerular PMN influx and glomerular damage during the anti-GBM glomerulonephritis model [3, 31]. Therefore, we wondered whether the sustained PMN presence, the transiently increased albuminuria and reduced renal function induced by co-injection of anti-HS antibodies, were mirrored into an enhanced fibrin deposition and hyalinosis. Two hours after induction of anti-GBM glomerulonephritis, glomeruli showed no visible signs of fibrin deposition in any of the experimental mice. After 1 day, mild fibrin deposition was detected in anti-GBM IgG-injected mice, whereas a significantly increased fibrin deposition was observed in mice co-injected with any of the four anti-HS antibodies (Fig 4A). However, after four days, fibrin deposition in anti-HS antibody co-injected mice hardly further increased, whereas fibrin deposition in mice injected with anti-GBM IgG alone did, resulting in a similar percentage of glomeruli with fibrin deposition for mice co-injected with anti-HS antibodies or not (Fig 4B). After 4 days, histological evaluation of the kidney tissues revealed diffuse glomerular injury (58%-98% affected glomeruli) in the anti-GBM injected mice, with or without co-injection of anti-HS antibodies (Fig 4C). The affected glomeruli showed prominent thrombosis and hyalinosis within the glomerular capillaries (Figs 4C and S3). We did not observe the formation of glomerular crescents or glomerulosclerosis. However, the tissues were sampled at day four after induction of our anti—GBM model, a time point at which the typical crescentic lesions are normally not present yet. Like the glomerular fibrin deposition, the observed thrombosis and hyalinosis may be indicative for the development of glomerular damage. In summary, co-injection of anti-HS antibodies initially results in an accelerated glomerular fibrin deposition, whereas 4 days after induction of anti-GBM glomerulonephritis, fibrin deposition as well as the presence of glomerular lesions appeared comparable among all treatment groups.

### Expression of pro-inflammatory mediators is increased in mice co-injected with anti-HS antibodies

**Fig 5:**
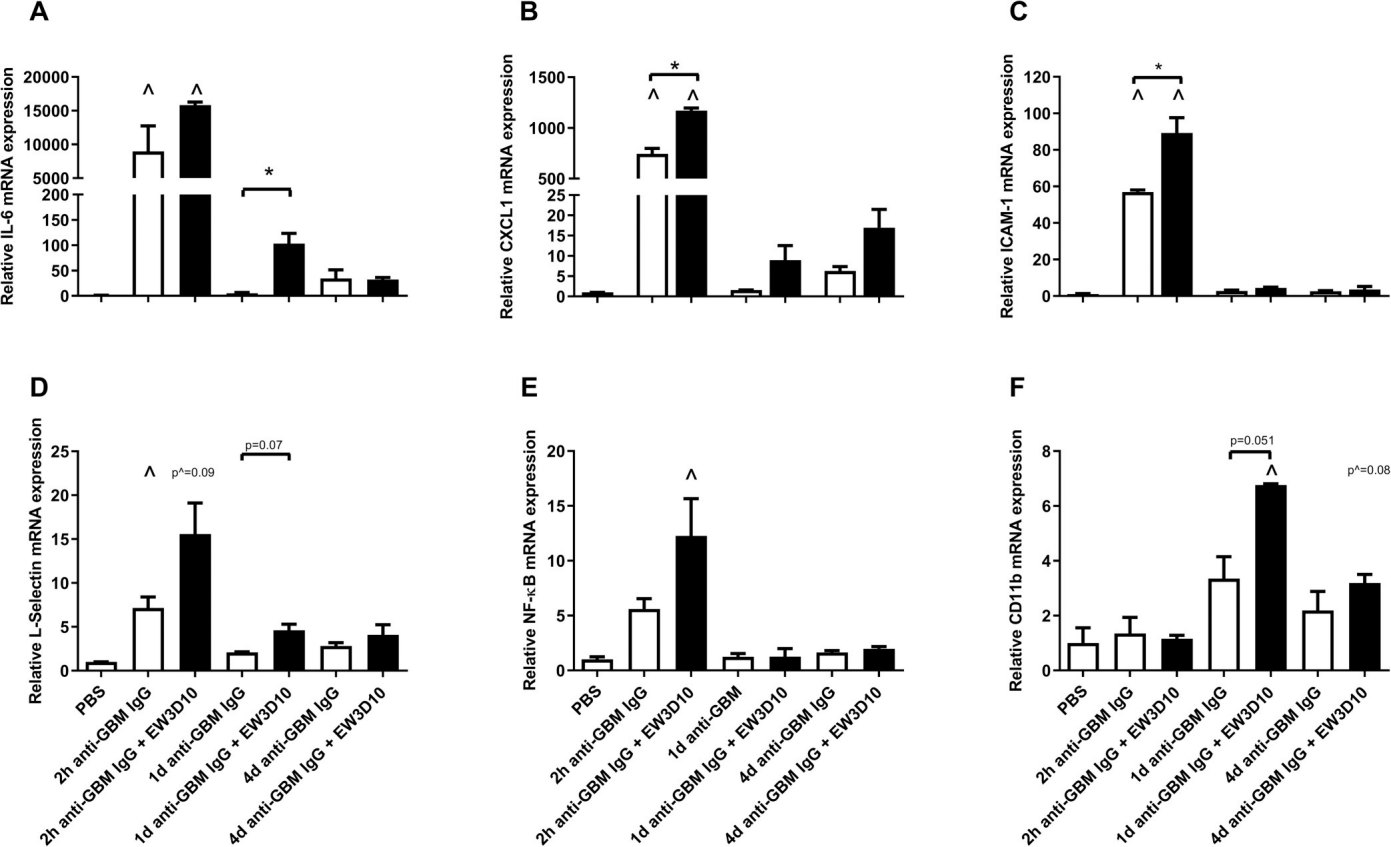
mRNA expression of pro-inflammatory mediators is increased after co-injection of anti-HS antibody EW3D10 in anti-GBM-induced glomerulonephritis. mRNA expression of (A) IL-6, (B) CXCL1, (C) ICAM-1, (D) L-selectin, (E) NF-ĸB, (F) CD11b in renal cortex from anti-GBM IgG- or EW3D10-co-injected mice after 2 hours, 1 day and 4 days. Graphs show the relative mRNA expression in experimental mice compared to PBS-treated mice, using GAPDH as the housekeeping gene. Results are expressed as means ± s.e.m. ^*P*<0.05 vs. PBS-injected mice. *P<0.05 between 2 groups.

To gain further insight into the observed anti-HS antibody-induced events, i.e. sustained PMN influx, renal mRNA expression of several pro-inflammatory mediators was analyzed in the mouse groups that, as a proof of concept, were co-injected with the anti-HS antibody EW3D10 or anti-GBM IgG alone. Following the trend of glomerular PMN influx, the expression of pro-inflammatory cytokine IL-6, the chemokine CXCL1, endothelial cell adhesion molecule ICAM-1, L-selectin and NF-κB was either significantly increased or showed a strong trend towards increased mRNA expression 2 hours after co-injection of anti-HS antibody EW3D10 compared to mice injected with anti-GBM IgG alone, which persisted up to 1 day after co-injection for IL-6, CXCL-1 and L-selectin (Fig 5A–5E). The expression of CD11b, the subunit of the integrin macrophage-1 antigen (Mac-1), was significantly increased 1 day after co-injection of anti-HS antibody EW3D10, compared to mice injected with anti-GBM IgG only (Fig 5F). The differences in mRNA expression of IL-6, CXCL1, L-selectin, ICAM-1 and NF-κB between the mouse groups co-injected with EW3D10 and those injected with anti-GBM IgG alone, became less apparent at later time points.

## Discussion

Previous studies designated the initial glomerular PMN influx as the main cause of renal damage and albuminuria in mice with anti-GBM-induced glomerulonephritis [3, 29]. Furthermore, we have shown *in vitro* and *in vivo* that specific highly sulfated HS domains recognized by anti-HS antibodies EW3D10, EW4G2 and AO4B08 are upregulated during inflammation [16]. Blocking of these HS domains *in vitro* with the respective antibodies resulted in differential inhibition of leukocyte adhesion, whereas the expression of other highly sulfated HS domains, e.g. detected with anti-HS antibody HS4C3, was not increased during inflammation, and also could not reduce leukocyte adhesion *in vitro* [16, 23]. Therefore, we postulated that blocking of specific pro-inflammatory HS domains, that serve as binding sites to PMNs, leads to inhibition of glomerular PMN influx, thereby protecting anti-GBM IgG-injected mice from developing glomerular injury and albuminuria. However, our present results show that glomerular PMN influx was not reduced after 2 hours by blocking inflammatory HS domains *in vivo* and albuminuria and renal function were not positively affected after 4 days of anti-GBM-induced glomerulonephritis. Unexpectedly, mice that were co-injected with anti-HS antibodies displayed partially transient effects which included: (i) a decreased renal function 2 hours and 1 day after induction of anti-GBM glomerulonephritis; (ii) an increased albuminuria 2 hours and 1 day after induction of anti-GBM glomerulonephritis; (iii) an increased glomerular fibrin deposition 1 day after induction of anti-GBM glomerulonephritis; (iv) a reduced glomerular macrophage influx 1 day after induction of anti-GBM glomerulonephritis and (v) a sustained glomerular presence of PMNs 1 day and 4 days after induction of anti-GBM glomerulonephritis, accompanied with an increased expression of IL-6, CXCL1, ICAM-1, L-selectin, CD11b, and NF-κB.

A possible explanation of the effects induced by anti-HS antibodies could be that the anti-HS antibodies affect the induction of the anti-GBM glomerulonephritis model. However, binding of rabbit anti-mouse GBM IgG and subsequent C3c deposition along the GBM was comparable in treatment groups. Therefore, it is unlikely that the anti-GBM glomerulonephritis model itself is affected by co-injection of anti-HS antibodies. We will discuss some possible mechanisms that may explain the observed effects induced by co-injection of anti-HS antibodies.

It is possible that specific binding of anti-HS antibodies to the glomerular endothelial glycocalyx during anti-GBM glomerulonephritis temporarily affects the integrity of the glycocalyx and/or GAG-ligand interactions. Previously, it was described that disruption of the endothelial glycocalyx by the HS-degrading enzyme heparanase can cause albuminuria as a consequence of disturbance of the negative charge barrier [1, 10, 32]. Since anti-HS antibodies contain a positively charged His-tag, it is possible that the negative charge in the glomerular filtration barrier (GFB) is partially neutralized by this tag or simply by binding of the anti-HS antibody to the HS domains within the GFB, thereby affecting barrier function and as a consequence induce a decrease of renal function and increase of albuminuria [1, 17]. However, control experiments in which only anti-HS antibodies were injected, did not shown any effect on albuminuria.

The anti-HS antibody-induced sustained glomerular presence of PMNs was not expected. Evaluation of inflammatory mediators revealed an increased mRNA expression of ICAM-1, NF-κB, IL-6, CXCL1, L-selectin and CD11b due to co-injection of anti-HS antibody EW3D10, compared to anti-GBM IgG alone. Regarding the increased mRNA expression of L-selectin after 2 hours in anti-HS antibody EW3D10 co-injected mice, one might argue that this is just a reflection of increased PMN influx due to co-injection. However, this increased L-selectin expression does not seem to correlate with the number of glomerular PMNs after 2 hours, which is comparable for both EW3D10-co-injected mice and mice injected with anti-GBM IgG alone. Notably, the increased CD11b mRNA expression after 1 day in EW3D10 co-injected mice also did not correlate with the number of glomerular macrophages at 1 day after injection. We postulate that co-injection of anti-HS antibodies results in endothelial activation, as reflected by increased NF-ĸB and ICAM-1 mRNA expression after 2 hours, and subsequent increase in inflammatory HS domains, enabling the binding of PMNs recruited in a IL-6- and CXCL1-dependent manner [16, 33–35]. The mechanism that underlies anti-HS antibody-induced endothelial cell activation remains unclear. However, recently, similar anti-HS antibodies were administrated during a study in glioblastoma tumors, resulting in a dose-dependent anti-HS antibody-mediated endothelial cell activation through p38 MAPK [36]. It may be possible that in our study, a similar mechanism of increased endothelial cell activation plays a role, thereby altering ligand (e.g. PMN) binding. Indeed, injections of EW3D10 alone, or combined with anti-GBM IgG, increase ICAM-1 mRNA expression two-fold compared to anti-GBM IgG alone, suggesting stronger endothelial activation upon anti-HS antibody injections. However, EW3D10 injections alone did not result in albuminuria, and equally increased BUN levels as did anti-GBM IgG injections alone. The complex and dynamic interplay between receptors and ligands on both PMNs and endothelial cells during experimental glomerulonephritis may be disturbed by anti-HS antibodies, thereby explaining the currently observed transient effects. Future research could be aimed at delineating specific receptor-ligand interactions, by targeting receptors, such as P-, E- and L-selectins, thereby evaluating the observed anti-HS antibody-mediated transient effects during experimental glomerulonephritis.

We furthermore suggest that the sustained presence of glomerular PMNs due to co-injection of anti-HS antibodies may be explained by the increased mRNA expression levels of L-selectin and CD11b after 1 day, compared to anti-GBM IgG injections alone. Another explanation of the anti-HS antibody-induced sustained glomerular presence of PMNs may be the blocking of HS domains on PMNs or endothelium by the applied antibodies, thereby affecting reversed migration of PMNs [37]. Surprisingly, during our study, the anti-HS antibody-induced sustained PMN presence does not contribute to eventual glomerular damage after 4 days anti-GBM-induced glomerulonephritis, since the final outcome regarding renal function, fibrin deposition, glomerular lesions and albuminuria is not affected by co-injection of anti-HS antibodies. Previous studies have shown that Mac-1 (consisting of CD11b and CD18)-deficient PMNs fail to induce proteinuria and Mac-1 appears essential for stabilizing PMN FcγR/IC interaction, enabling firm adhesion to the endothelial cells. Also, interaction between Mac-1 and complement C3bi, which includes the C3c fragment, appears to be required for the release of azurophilic granules leading to proteinuria [38]. Given the fact that HS is a Mac-1 ligand which enhances the binding of leukocytes, it is possible that anti-HS/EW3D10 binding on PMNs and/or endothelia impairs correct integrin clustering, Mac-1 dependent stabilization of PMN FcγR/IC interaction and/or Mac-1 interaction with C3bi. This might subsequently lead to impaired PMN activation, impaired azurophilic granule release and/or impaired oxidative burst, resulting in the absence of further kidney damage and albuminuria [7, 33, 38–42]. This might explain why after 4 days of anti-GBM-induced glomerulonephritis, despite sustained PMN presence, fibrin deposition, glomerular lesions, albuminuria and renal function are comparable in mice that were co-injected with anti-HS antibodies compared to mice with anti-GBM-induced glomerulonephritis only.

We conclude that, contrary to our hypothesis, blocking of the inflammatory HS domains with anti-HS antibodies does not lead to a reduced glomerular PMN influx during anti-GBM-induced glomerulonephritis, but rather a sustained glomerular presence of PMNs. Moreover, co-injection of anti-HS antibodies transiently worsens albuminuria and renal function. However, despite the sustained presence of PMNs, co-injection of anti-HS antibodies during anti-GBM-induced glomerulonephritis does not affect the final outcome regarding albuminuria and renal function.

We conclude that the evaluated anti-HS antibodies do not show therapeutic potential against anti-GBM-induced glomerulonephritis. Future research should evaluate other strategies to target HS domains involved in inflammatory processes during glomerulonephritis.

## Supporting information

S1 Fig Immunofluorescence staining of PMNs in glomeruli of control and anti-GBM IgG injected mice. Representative immunofluorescence stainings for PMNs with GR-1 antibody (green) and anti-agrin co-staining (red), 2 hours, 1 day and 4 days after injection with PBS, anti-GBM IgG and anti-GBM IgG + scFv. White arrowheads indicate the presence of PMNs in the glomeruli.
(TIF)

S2 Fig Immunofluorescence staining of macrophages in glomeruli of control and anti-GBM IgG injected mice. Representative immunofluorescence stainings for macrophages with anti-CD68 antibody (green) and anti-agrin co-staining (red), 2 hours, 1 day and 4 days after injection with PBS, anti-GBM IgG and anti-GBM IgG + scFv. White arrowheads indicate the presence of macrophages in the glomeruli.
(TIF)

S3 Fig Representative images of periodic acid-Schiff and hematoxylin stained renal sections. Images illustrate what was scored as normal glomeruli, < 50% affected glomeruli, or >50% affected glomeruli.
(TIF)
