# Supplementary figures and images for: Blocking of inflammatory heparan sulfate domains by specific antibodies is not protective in experimental glomerulonephritis
Source: PLoS One. 2021 Dec 23;16(12):e0261722. doi: 10.1371/journal.pone.0261722 (PMC8699719; doi:10.1371/journal.pone.0261722)

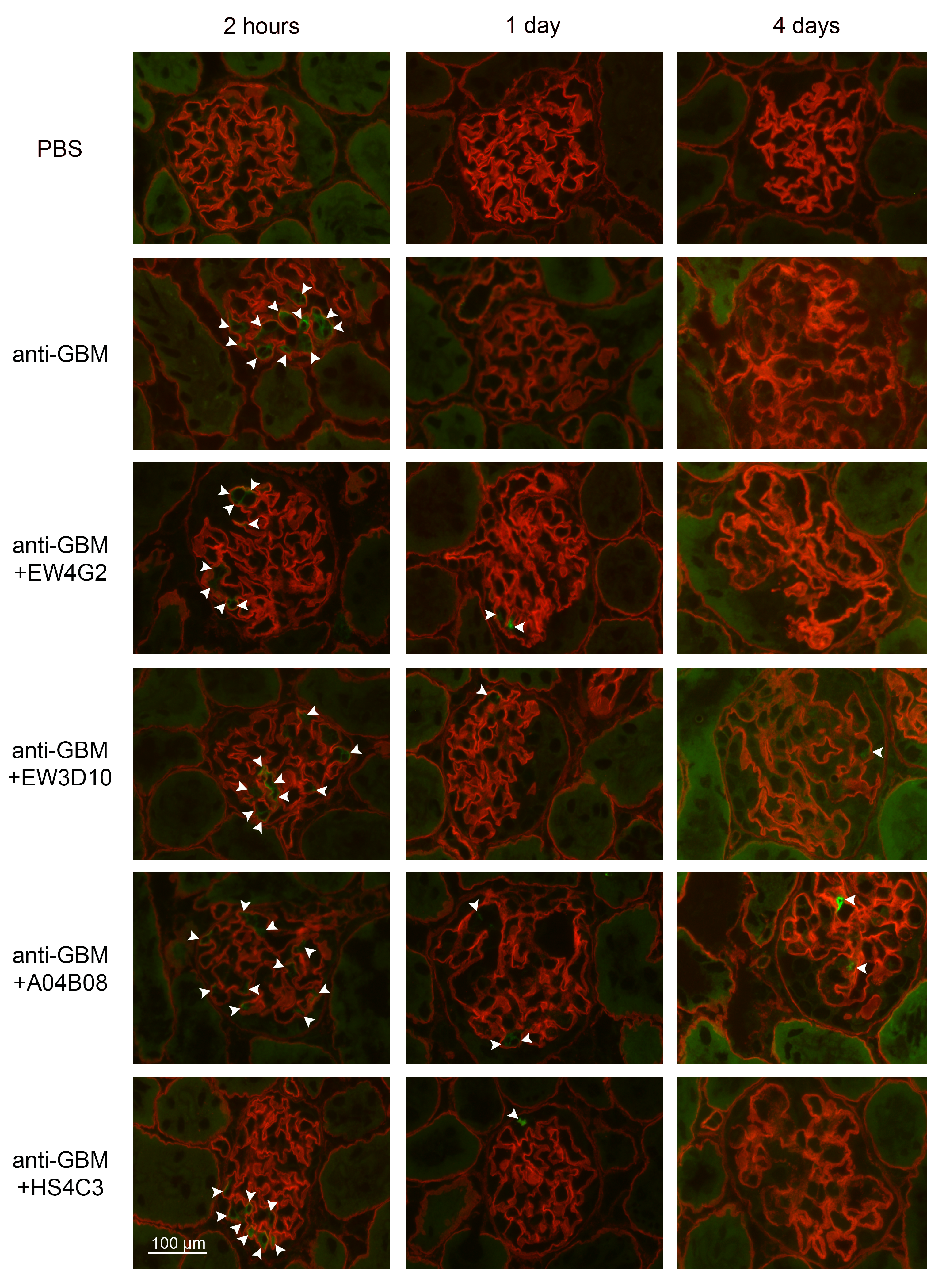

Supplement: S1 Fig — Representative immunofluorescence stainings for PMNs with GR-1 antibody (green) and anti-agrin co-staining (red), 2 hours, 1 day and 4 days after injection with PBS, anti-GBM IgG and anti-GBM IgG + scFv. White arrowheads indicate the presence of PMNs in the glomeruli. (TIF) [file pone.0261722.s001.tif]

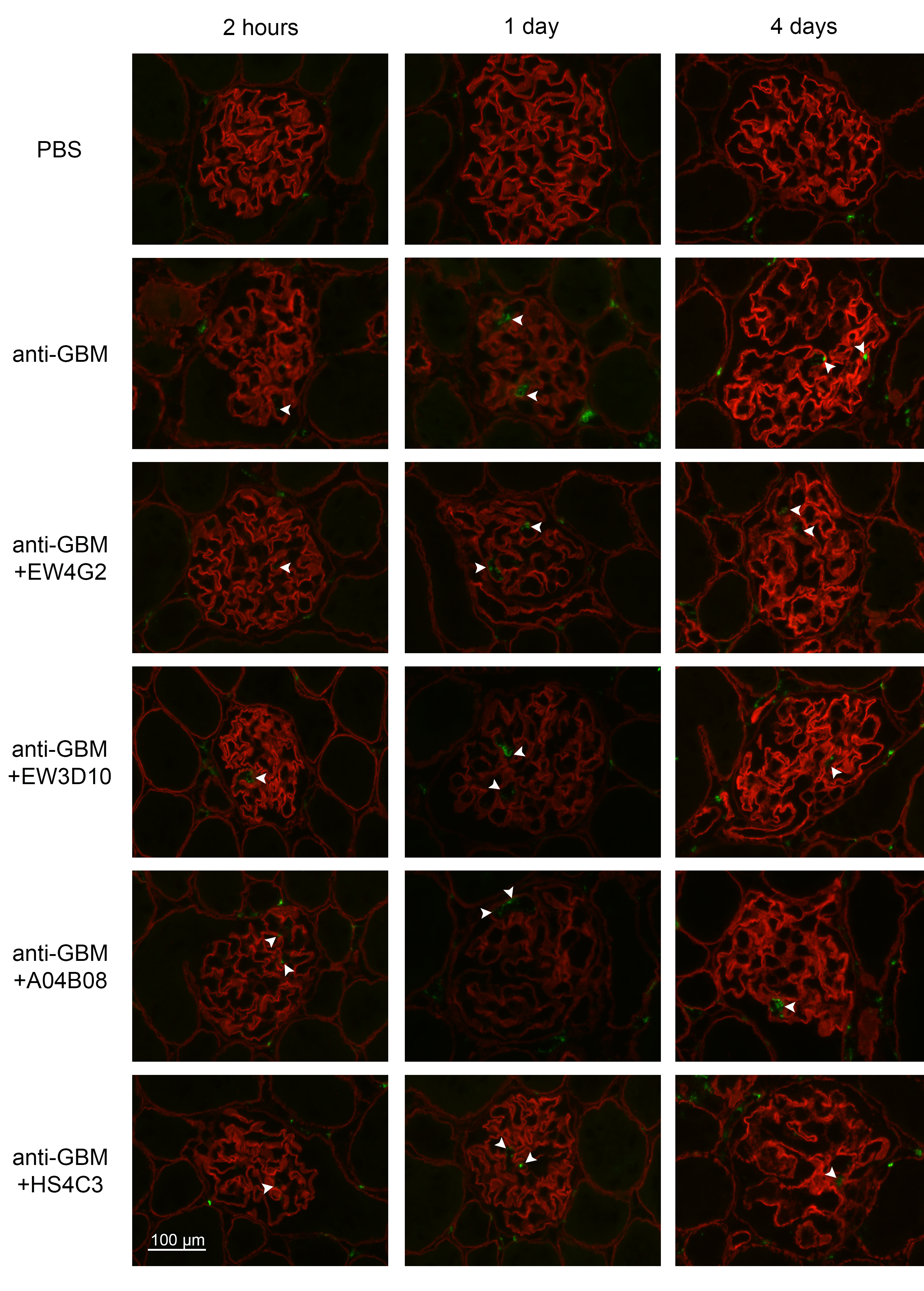

Supplement: S2 Fig — Representative immunofluorescence stainings for macrophages with anti-CD68 antibody (green) and anti-agrin co-staining (red), 2 hours, 1 day and 4 days after injection with PBS, anti-GBM IgG and anti-GBM IgG + scFv. White arrowheads indicate the presence of macrophages in the glomeruli. (TIF) [file pone.0261722.s002.tif]
